# Ground-State Structure of Quaternary Alloys (SiC)_1−*x*_ (AlN)*_x_* and (SiC)_1−*x*_ (GaN)*_x_*

**DOI:** 10.3390/mi14020250

**Published:** 2023-01-19

**Authors:** Abdelkader Menad, Mohamed Ferhat, Ali Zaoui

**Affiliations:** 1 Département de Génie Physique, (LPMF), Université des Sciences et de la Technologie d’Oran, Mohamed Boudiaf, El Mnaouar, BP 1505, Bir El Djir, Oran 31000, Algeria; 2 Département de Physique, Université Oran 1, Ahmed Ben Bella, BP 1524, El M’Naouer, Oran 31000, Algeria; 3 Laboratoire de Génie Civil et géo-Environnement, Univ. Lille, IMT Nord Europe, JUNIA, Univ. Artois, ULR 4515-LGCgE, F-59000 Lille, France

**Keywords:** quaternary alloys, (SiC)_x_(AlN)_1−x_, (SiC)_x_(GaN)_1−x_, DFT, evolutionary algorithms

## Abstract

Despite III-nitride and silicon carbide being the materials of choice for a wide range of applications, theoretical studies on their quaternary alloys are limited. Here, we report a systematic computational study on the electronic structural properties of (SiC)*_x_* (AlN)_1−*x*_ and (SiC)*_x_* (AlN)_1−*x*_ quaternary alloys, based on state-of-the-art first-principles evolutionary algorithms. Trigonal (SiCAlN, space group *P*3m1) and orthorhombic (SiCGaN, space group *P*mn2_1_) crystal phases were as predicted for *x* = 0.5. SiCAlN showed relatively weak thermodynamic instability, while that of SiCGaN was slightly elevated, rendering them both dynamically and mechanically stable at ambient pressure. Our calculations revealed that the *P*m31 crystal has high elastic constants, (*C*_11_~458 GPa and *C*_33_~447 GPa), a large bulk modulus (*B*_0_~210 GPa), and large Young’s modulus (*E*~364 GPa), and our results suggest that SiCAlN is potentially a hard material, with a Vickers hardness of 21 GPa. Accurate electronic structures of SiCAlN and SiCGaN were calculated using the Tran–Blaha modified Becke–Johnson semi-local exchange potential. Specifically, we found evidence that SiCGaN has a very wide direct bandgap of 3.80 eV, while that of SiCAlN was indirect at 4.6 eV. Finally, for the quaternary alloys, a relatively large optical bandgap bowing of ~3 eV was found for SiCGaN, and a strong optical bandgap bowing of 0.9 eV was found for SiCAlN.

## 1. Introduction

Materials known as III-V binary nitrides (III-N = AlN, SiC, and GaN) [1,2,3,4,5,6,7,8,9,10,11] and their related alloys have recently drawn attention owing to their outstanding optoelectronic properties making them useful for many practical applications, such light-emitting diode lasers (LEDs). The reasons for their successful application are their plethora of physical characteristics, such as their small lattice parameter, large direct bandgap, high hardness, high temperature stability, good piezoelectric properties, and polytypism. The group-III nitrides crystallize as a würtzite (WZ, 2H) structure under ambient conditions, and can be also grown in a cubic zinc blende (ZB, 3C) phase.

As an analog to nitride-based semiconductors, silicon carbide (SiC), the only stable compound of the IV-IV family, is a wide bandgap material, and it exhibits more than 250 polytypes (in particular, SiC adopts the cubic (3C) and the würtzite hexagonal (2H) form). Moreover, SiC has particular properties that make it suitable for use in high-power, high-temperature, and high-speed microelectronic device applications.

Interestingly, making solid solutions from alloys of SiC and III-N compounds offers excellent options for optoelectronic engineering applications, as this enhances the functionalities of the SiC and III-N compounds, and not only exploits the difference in bandgap (*E*_G_) in order to tailor the *E*_G_ and other properties, but may also produce exceptionally hard coatings.

Typically, SiCAlN solid solutions have often been synthetized at high temperatures using magnetron sputtering techniques [12,13]. Thin-film SiCAlN solid solutions with low temperature growth have been obtained via gas-source molecular beam epitaxy (GSMBE) [14] and MBE [15,16] methods. Moreover, there is a limited amount of research dedicated to SiCGaN and SiCAlN quaternary alloy systems [15,17,18,19].

All previous works [15,17,18,19] have used first-principle methods and small supercells of würtzite and/or cubic structures. Such an approach can, in principle, give adequate results for alloys containing constituents of small size and chemical mismatch, however, for large mismatched alloys, very large supercells are required to adequately reproduce the electronic properties of alloys (i.e., a large number of atoms, such as 64 or 128, is needed). Moreover, these methods require a large number of atoms in order to attain statistical significance. However, there is a crucial problem concerning the global stability of the hypothetical phase of the alloys designated by the small/large supercell. Generally, this phase is erroneously taken for granted (i.e., the proposed structure is often considered stable). The routine calculations [15,17,18,19] give the wrong structure when their selection is based upon a few estimated ‘usual-suspect-phases’. The determination of the electronic structural properties is largely irrelevant if the hypothetical phases of the alloy are not globally stable—this includes their thermodynamic, mechanical, and, more importantly, dynamic stabilities. These phases could have exciting and interesting optoelectronic properties, but equally they could not exist at all. 

In the present study, we used an attractive paradigm approach; the global evolutionary structure optimization (GESO) method was implemented using the high-throughput Open Quantum Materials Database (OQMD) [20,21]. The GESO avoids the problems associated with a single starting structure. The prediction of stable crystal structures based only on the knowledge of the chemical composition has long remained a major unsolved problem in the condensed matter physics of crystalline solids. In this context, the GESO approach was targeted in order to find the most stable crystalline structure for a given chemical composition based on the concepts of the Darwin evolutionary theory. 

In the present work, using a first-principles structure search, we explored the atomic and electronic structural properties of (SiC)*_x_*(AlN)_1−*x*_, and (SiC)*_x_*(GaN)_1−*x*_ quaternary compounds. A detailed investigation was performed to elucidate their phase stability and their mechanical and electronic properties. To further improve their optoelectronic properties, their bandgap composition dependence was also investigated.

## 2. Computational Methods

Searches to discover the ground state phases in the (SiC)*_x_*(III-N)_1−*x*_ systems were performed using the evolutionary algorithm, as implemented in the Material Project Database [20,21]. Structural optimizations and calculations of total energies were carried out within the framework of a density functional theory (DFT) approach [22], as implemented in the Plane-Wave Quantum ESPRESSO package [23]. The electron exchange–correlation effects were approximated using the local density approximation (LDA) [24], and the projector augmented plane wave (PAW) approach [25] was used to describe the electron–ion interactions. The electronic wave functions were expanded in plane waves up to an energy cutoff of 60 Ry. Integrations over the Brilloun zone were sampled with a 8 × 6 × 6 Monkhorst-Pack [26] *k*-points grid. Full relaxations of lattice parameters and atomic positions were performed until forces acting on an atom did not exceed 10^−4^ eV/Å, and crystal total energy was converged to 10^−5^ eV /cell.

The phonon calculations were performed using the density functional perturbation theory (DFTP) [27]. The electronic properties were studied using all electron code WIEN2k [28], which is based on the full-potential linearized augmented plane wave (FP-LAPW) method. The product of the smallest atomic spheres radius, *R*_MT_, multiplied by the largest *K*-vector, *K*_max_ (*R*_MT_*K*_max_) was selected as 8. The muffin-tin radius for each of the Al, Ga, C, Si, and N atoms was chosen to be 1.71, 1.67, 1.51, and 1.63 au, respectively, for SiCAlN, and 1.62, 1.61, 1.54, and 1.40 au, respectively, for SiCGaN. 

The Kohn–Sham DFT within the LDA or GGA has been proven to be efficient in computing the structural properties of materials. However, it is well known that the standard LDA/GGA procedure severely underestimates band gaps, typically by 50 to 100% (the so-called ‘band gap problem’). Several methods for overcoming this limitation have been proposed. One of them is the modified Becke–Johnson exchange potential (TB-mBJ) [29]. The TB-mBJ can give remakably acurrate band gaps for a variety of solids, inclduing wide band gap insulators, sp-semiconductors, and strongly correlated 3d transition metals [29], and it competes in accuracy with the more expensive hybrid and GW methods. In this context, we used the Tran–Blaha modified Becke–Johnson (TB-mBJ) semi-local exchange potential to study the electronic properties of SiCAlN and SiCGaN quaternary systems.

## 3. Results and Discussion

The ground-state lattice parameters of the WZ-2H stable structures of the binary III-N compounds are listed in Table 1. The calculated lattice constants and *c*/*a* ratio were in excellent agreement with experimental measurements, being within 1% and 0.2% of the experimental data, respectively [30,31,32,33].

**Figure 1:**
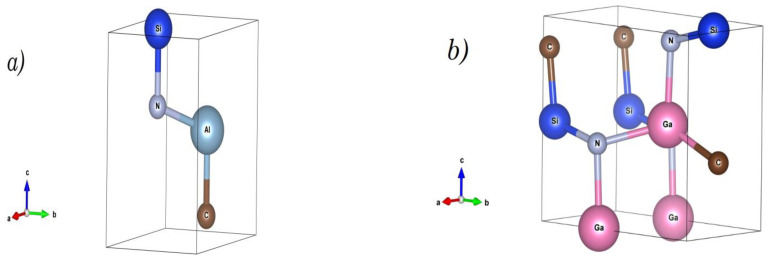
The trigonal (*P*3M1) and orthorhombic (*P*mn2_1_) crystal structures of (**a**) SiCAlN and (**b**) SiCGaN quaternary alloy systems.

The ground-state searches (for *x* = 0.5) produced two low enthalpy structures for the (SiC)*_x_*(III-N)_1−*x*_ quaternary alloys (Figure 1), namely trigonal SiCAlN (space group, *P*3m1, N° = 156), and SiCGaN, which adopts the orthorhombic structure (space group *P*mn2_1_, N° = 31). The calculated optimized crystalline parameters of SiCAlN and SiCGaN are listed in Table 2.

**Figure 2:**
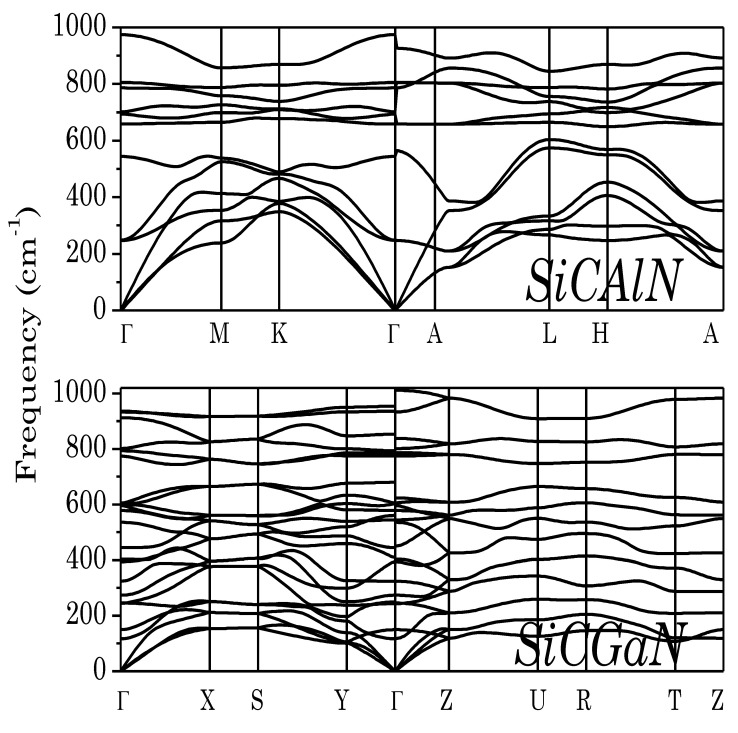
Phonon band structure of SiCAlN, and SiCGaN quaternary alloy systems.

Next, to ensure that the predicted ground-state phases of SiC–III-N compounds were dynamically stable, we calculated phonon dispersion curves (Figure 2). The calculated phonon dispersion curves had no soft modes in the entire Brillouin zone, demonstrating the dynamical stabilities of the above phases.

In order to predict whether, and under which conditions, these quaternary alloys can be synthesized, we assessed the feasibility of doping SiC in AlN and GaN compounds. The alloy formation enthalpy is defined for *x* = 0.5 as:

$$\Delta H=E_{total}\left(SiC-IIIN\right)-E_{total}\left(SiC\right)-E_{total}\left(III-N\right)$$

We found that Δ*H* was 67 and 182 meV/atom, respectively, for the SiCAlN and SiCGaN quaternary alloys. The positive values of Δ*H* indicate the tendency for phase separation into binary compounds as relatively weak for SiCAlN, and relatively strong for SiCGaN. Our Δ*H* value for SiCAlN was consistent with previous reports [15], where Δ*H* was found to be quite small (Δ*H* ~50 meV/atom). However, to prevent phase separation at finite temperatures, the metastable phases of SiCAlN and SiCGaN can be stabilized through entropy. In a regular solution, the miscibility gap temperature is given by $T_{mis}=\frac{2\Delta H}{K_{R}}$, where *k*_B_ is the Boltzmann constant. We found that the critical temperature, $T_{mis}$, above which complete miscibility is possible, was 1282 and 4224 °C for SiCAlN and SiCGaN, respectively. Our value for SiCAlN is consistent with previously reported experimental results, where researchers typically used MBE and GMBE grow techniques with temperatures of 750–1300 °C [14,15]. For SiCGaN, the results suggest that (SiC)*_x_*(Ga-N)_1−*x*_ quaternary alloys could be stable over a wide range of moderate compositions of *x* at normal growth temperatures for poor (SiC) or rich (GaN) compositions. Unfortunately, no experimental measurements are available for SiCGaN. To understand the thermodynamic instability of SiCAlN and SiCGaN, we decomposed the formation enthalpy Δ*H* into two individual physical mechanisms [34], expressing it as a sum of structural (St) and chemical (Chem) contributions: ΔH=ΔH(St)+ΔH(Chem).

Table 3 lists the decomposition results for SiC–III-N quaternary alloys. The results reveal that the structural energy ΔH(St) in SiCAlN and SiCGaN is relatively small, and hence mixing is encouraged. The calculated weak ΔH(St) correlates with the small lattice mismatch $\frac{\Delta a}{a}$ between the SiC and III-N compounds: $\frac{\Delta a}{a}=$ 1–3% (SiCAlN, SiCGaN).

Our results above lead us to the conclusion that the large positive chemical energy Δ*H*(Chem) contribution to Δ*H* is the key factor that is controlling the instability of SiCAlN and SiCGaN quaternary alloys.

The elastic constants (*C*_ij_) of the *P*3m1 and *P*mn2_1_ crystal phases were evaluated to verify their mechanical stabilities (Table 4). All materials studied were found to be mechanically stable—the entire set of the elastic constants *C*_ij_ satisfied the elastic Born–Hung criteria [35]. Moreover, we found that SiCAlN possesses high elastic constants (*C*_11_~458 GPa, and *C*_33_~447 GPa), indicating that the *P*3m1 crystals exhibit excellent resistance to deformation along the *a*- and *c*-axes. The Young’s modulus, *E*, the shear modulus, *G,* and the Poisson’s ratio, *v*, derived from the calculated *C*_ij_ values, are listed in Table 5. The calculated values of *B* and *G* for SiCAlN were ~216 GPa and ~149 GPa, respectively, indicating the strongly incompressible nature of the *P*3m1 phase. The *B*/*G* ratio characterizes the ductile ($\frac{B}{g}>1.75)$ versus brittle ($\frac{B}{g}<1.75$) nature of materials. SiCGaN exhibited ductile characteristics ($\frac{B}{G}=2.04)$, whereas SiCAlN showed some degree of brittleness ($\frac{B}{G}=1.45)$. 

We then estimated the hardness of SiC–III-N compounds using the Microscopic Chen’s model [36]: $H_{v}=2{\left[\frac{G^{3}}{B^{2}}\right]}^{0.585}-3$. The hardness of SiCAlN and SiCGaN at ambient conditions was 21.2 GPa and 8.5 GPa, respectively, suggesting the *P*3m1 crystal is potentially a hard material.

**Figure 3:**
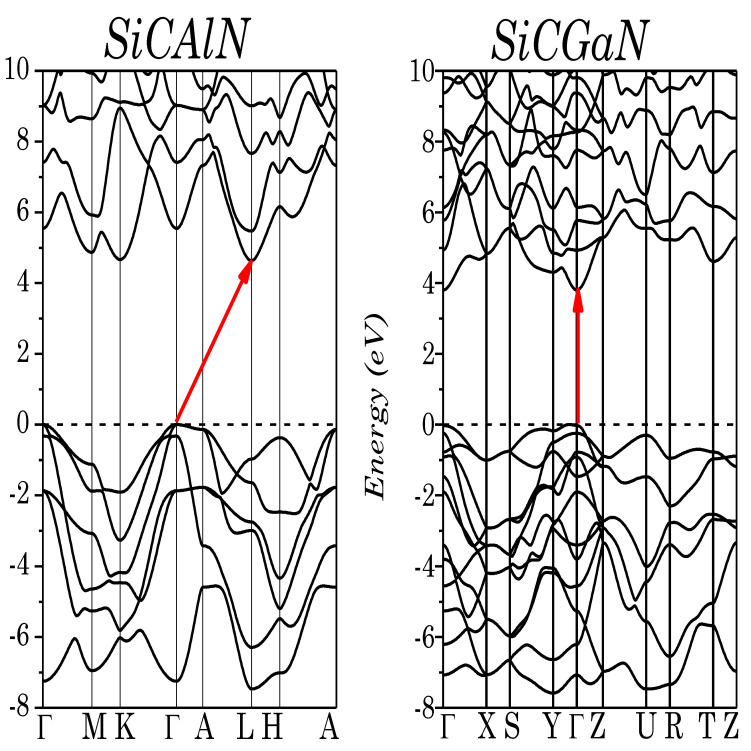
Electronic band structure of SiCAlN, and SiCGaN quaternary alloy systems.

The electronic-mBJ band structure of the trigonal (SiCAlN) and orthorhombic (SiCGaN) phases are shown in Figure 3. SiCGaN has a wide direct bandgap (Γ → Γ) of 3.80 eV, whereas SiCAlN has a very wide indirect band gap (Γ → L) of 4.64 eV. Various calculations have been performed for this system, for instance Roucka et al. [15], using a plane-wave pseudopotential method within the GGA, found a lower bandgap of 3.2 eV, whereas Tang et al. [17], using first-principle pseudopotential calculations within the LDA, found a bandgap of 2.6 eV. All of these calculations used approximations (LDA/GGA) which are known to severely underestimate the excited states (i.e., bandgaps). However, no theoretical or experimental data are available for SiCGaN.

These electronic properties render SiC–III-N quaternary alloys as appropriate transparent compounds for use as the window layer in solar cell applications (i.e., they are materials with bandgap *E*_G_ > 3 eV). Note that during the growth of wide band gap binaries such as III-N and SiC, some defects [37,38] can be created, which originate from the atomic layout imperfection caused by growth temperature fluctuation or strain. These intrinsic defects, including vacancies and their associated complexes, have different charge configurations, and can be located in the forbidden band, which can directly impact the electronic properties of the host material. It should be noted that the current DFT-based investigation of AlN, GaN, SiC and their related quaternary systems was conducted without taking into account the presence of defects—the materials studied were considered to be ideal crystal structures. This question will be addressed and explored in depth in the near future as part of a separate study. 

**Figure 4:**
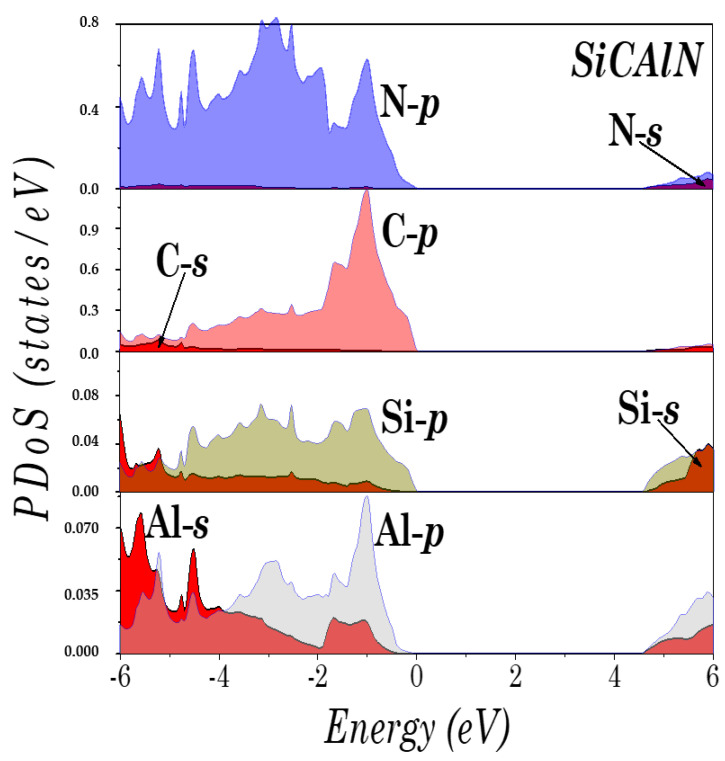
Partial density of states (DoS) of SiCAlN.

**Figure 5:**
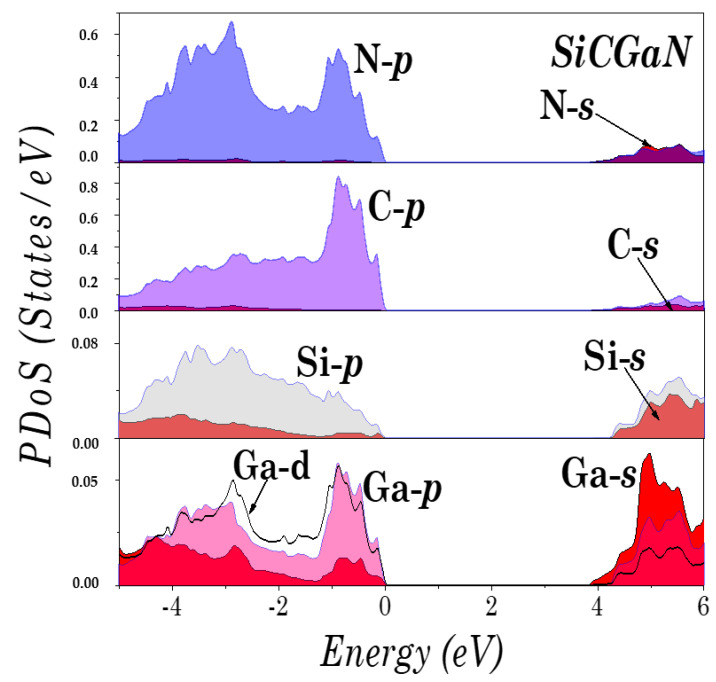
Partial density of states (DoS) of SiCGaN.

To understand the electronic structure of the studied compounds, the partial density of states (PDoS) of SiCAlN and SiCGaN are illustrated in Figure 4 and Figure 5. It was found that the top of the valence bands originates mainly from the coupling of the Al-*p* (Ga-*d*, and Ga-*p*) and N-*p* states, and the Si and C *p* states. Note also, the unusual ‘bad’ bonding states (i.e., where the total number of valence electrons deviates by $Z_{v}=-1$ from the normal octet, such in the ‘normal’ Ga-N bond), induced by the interaction between the Al-*p* (Ga-*d*, and Ga-*p*) and C-*p* states, and between the Si-*p* and N-*p* states.

Finally, the calculated band gaps of the III-N compounds were used along with the calculated values of SiC–III-N to obtain the variation in the direct energy gap of the quaternary alloys:

$$E_{G}\left(x\right)=\left(1-x\right)E_{G}\left(III-N\right)+xE_{G}\left(SiC\right)-bx\left(1-x\right)$$

where *b* is known as the bowing parameter. We found, for *x* = 0.5, a relatively large optical bandgap bowing of *b* = 0.9 eV for SiCAlN, and a strong optical bandgap bowing of 2.98 eV for SiCGaN. 

Our calculated bandgap bowing value for SiCAlN is much lower than both the measured value of ~2.7 eV [12] and the DFT-calculated values of ~6 eV [17] and ~8.8 eV [15]. However, no theoretical or experimental data are available for SiCGaN. 

## 4. Conclusions

In summary, we explored the electronic structural properties of (SiC)*_x_* (III-N)_1−*x*_ quaternary alloys based on structure searching and density functional theory methods. Two ordered trigonal (SiCAlN, space group *P*3m1) and orthorhombic (SiCGaN, space group *P*mn2_1_) phases were determined for *x* = 0.5. The thermodynamic stability calculations evinced that SiCAlN has a weak formation enthalpy of 67 meV/atom, and that that of SiCGaN is moderate at 183 meV/atom).

The results showed that the *P*3m1 and *P*mn2_1_ crystal phases are dynamically and mechanically stable at ambient conditions, as determined by examining the phonon spectra and elastic constants. Furthermore, SiCAlN was found to potentially be a hard material with strong elastic constants (*C*_11_~458 GPa, and *C*_33_~447 GPa), a high bulk modulus (*B*_0_~210 GPa), a Young’s modulus of *E*~364 GPa, and a Vickers hardness of 21 GPa.

Analysis of the electronic properties demonstrated that SiCGaN is a wide direct bandgap semiconductor, measured at 3.80 eV, and that SiCAlN is an indirect bandgap semiconductor, measured at 4.60 eV. Moreover, for these quaternary alloys, a relatively large optical bandgap bowing of *b* = 0.9 eV was found for SiCAlN, and a strong one of 2.98 eV was found for SiCGaN.

## Figures and Tables

**Table 1:** The calculated equilibrium structural parameters, bulk modulus (*B_0_*), and its pressure derivative (*B’_0_*) of WZ AlN, GaN and SiC compounds, and the available experimental data [30,31,32,33].

	*a* (Å)	*c*/*a*	*B_0_* (GPa)	*B’_0_*
**AlN**	3.087 (3.112) ^a^	1.600 (1.600) ^a^	210.5 (185) ^b^	3.1
**GaN**	3.160 (3.189) ^a^	1.630 (1.626) ^a^	184.7 (188) ^b^	4.2
**SiC**	3.055 (3.079) ^c^	1.641 (1.641) ^c^	145.6 (223) ^d^	3.8

^a^: ref. [30], ^b^: ref. [31], ^c^: ref. [32], ^d^: ref. [33].

**Table 2:** The calculated equilibrium structural parameters, bulk modulus (*B_0_*), and its pressure derivative (*B’_0_*) of trigonal (*P*3m1) and orthorhombic (*P*mn2_1_) crystals.

	*a* (Å)	*b*/*a*	*c*/a	*B_0_* (GPa)	*B’_0_*
SiCAlN(*P*3m1)	3.071		1.625	216.4	3.9
SiCGaN(*P*mn2_1_)	3.162	1.840	1.658	170.5	3.2

**Table 3:** Calculated structural energy of formation Δ*H*(St), chemical energy of formation Δ*H*(Chem), and total energy of formation Δ*H*.

	Δ*H*(St) (eV/atom)	Δ*H*(Chem) (eV/atom)	Δ*H* (eV/atom)
SiCAlN	−0.087	0.155	0.067
SiCGaN	0.046	0.136	0.183

**Table 4:** Calculated elastic constants *C*_ij_ in GPa for *P*3m1 and *P*mn2_1_ crystal phases.

	*C*_11_	*C*_12_	*C*_22_	*C*_13_	*C*_23_	*C*_33_	*C*_44_	*C*_55_	*C*_66_
SiCAlN	457.9	125.4		86.5		447.2			166.3
SiCGaN	356.3	84.2	156.8	122.4	153.0	331.1	131.6	106.9	69.6

**Table 5:** Calculated Young’s modulus *E*, shear modulus *G*, and Poisson’s ratio *ν* for *P*3m1 and *P*mn2_1_ crystal phases.

	*E* (GPa)	*G* (GPa)	*ν*
SiCAlN	364.3	149.2	0.22
SiCGaN	212.9	83.5	0.28

## Data Availability

The data of this work are available from the corresponding author upon reasonable request.
